# A New Drug Discovery Platform: Application to DNA Polymerase Eta and Apurinic/Apyrimidinic Endonuclease 1

**DOI:** 10.3390/ijms242316637

**Published:** 2023-11-23

**Authors:** Debanu Das, Matthew A. J. Duncton, Taxiarchis M. Georgiadis, Patricia Pellicena, Jennifer Clark, Robert W. Sobol, Millie M. Georgiadis, John King-Underwood, David V. Jobes, Caleb Chang, Yang Gao, Ashley M. Deacon, David M. Wilson

**Affiliations:** 1 XPose Therapeutics, Inc., San Carlos, CA 94070, USA; 2 Accelero Biostructures, Inc., San Carlos, CA 94070, USA; 3 Mitchell Cancer Institute and Department of Pharmacology, University of South Alabama, Mobile, AL 36604, USA; 4 Department of Pathology & Laboratory Medicine, Warrant Alpert Medical School & Legorreta Cancer Center, Brown University, Providence, RI 02912, USA; 5 Department of Biochemistry and Molecular Biology, Indiana University School of Medicine, Indianapolis, IN 46202, USA; 6 Mid-Atlantic BioTherapeutics, Inc., Doylestown, PA 18902, USA; 7 Department of BioSciences, Rice University, Houston, TX 77251, USA; 8 Biomedical Research Institute, Hasselt University, 3500 Diepenbeek, Belgium; 9 Belgium & Boost Scientific, 3550 Heusden-Zolder, Belgium

**Keywords:** fragment-based drug discovery, structure-based drug discovery, X-ray crystallography, cancer therapeutics, DNA damage response, polymerases, Pol eta, POLH, APE1, targeted protein degradation, synthetic lethality

## Abstract

The ability to quickly discover reliable hits from screening and rapidly convert them into lead compounds, which can be verified in functional assays, is central to drug discovery. The expedited validation of novel targets and the identification of modulators to advance to preclinical studies can significantly increase drug development success. Our SaXPy^TM^ (“SAR by X-ray Poses Quickly”) platform, which is applicable to any X-ray crystallography-enabled drug target, couples the established methods of protein X-ray crystallography and fragment-based drug discovery (FBDD) with advanced computational and medicinal chemistry to deliver small molecule modulators or targeted protein degradation ligands in a short timeframe. Our approach, especially for elusive or “undruggable” targets, allows for (i) hit generation; (ii) the mapping of protein–ligand interactions; (iii) the assessment of target ligandability; (iv) the discovery of novel and potential allosteric binding sites; and (v) hit-to-lead execution. These advances inform chemical tractability and downstream biology and generate novel intellectual property. We describe here the application of SaXPy in the discovery and development of DNA damage response inhibitors against DNA polymerase eta (Pol η or POLH) and apurinic/apyrimidinic endonuclease 1 (APE1 or APEX1). Notably, our SaXPy platform allowed us to solve the first crystal structures of these proteins bound to small molecules and to discover novel binding sites for each target.

## 1. Introduction

Cancer will directly affect the lives of over one-third of the global population. The process of carcinogenesis involves (at least) six biological hallmarks [1]: sustaining proliferative signaling, evading growth suppressors, resisting cell death, enabling replicative immortality, inducing angiogenesis, and activating invasion and metastasis. Many, if not all, of these hallmarks can be attributed to genomic instability that arises from excessive DNA damage or defects in the DNA damage response (DDR) components. DDR is an intricate system, which involves damage recognition, DNA damage repair, cell cycle regulation, and cell fate determination, playing a prominent role in cancer etiology and therapy [2]. DDR pathways are frequently upregulated in cancer cells as a compensatory mechanism to adapt to elevated background levels of DNA damage from rapid cell division and increased metabolism [3] or genotoxic stress induced by many anti-cancer agents, including radiotherapy and certain forms of chemotherapy [4,5]. The realization that such intrinsic changes in the DDR (i.e., sporadic inactivation or upregulation) offer therapeutic opportunities has led to advances in cancer treatment efficacy.

The discovery that homologous recombination repair (HRR)-defective breast and ovarian cancers are uniquely sensitive to poly-(ADP-ribose) polymerase (PARP) inhibitors has driven the renewed interest in the design of DDR inhibitors to combat both intrinsic, cancer-specific characteristics and acquired drug resistance [6,7]. Exploiting such so-called synthetic lethality (SL), where PARP inhibitors drive the accumulation of cytotoxic DNA intermediates that are normally resolved via HRR, has motivated improved drug design/application and has led to better outcomes for many of these cancer-affected individuals [8]. Moreover, developed drugs will widen the repertoire of initial treatment options and have potential utility in re-sensitizing cells to genotoxic therapies that have failed due to the upregulation of DDR pathways. Thus, several efforts around the world are focused on the development of novel small molecule inhibitor(s) (SMI) or targeted protein degradation (TPD) cancer therapeutics against DDR proteins, with an eye on SL and combinatorial treatment opportunities [9]. Here, we give a brief overview of some current drug discovery efforts before presenting a novel strategy, termed SaXPy (SAR by X-ray Poses Quickly), and its application to two DDR proteins, DNA polymerase eta (Pol η or POLH) and apurinic/apyrimidinic endonuclease 1 (APE1 or APEX1).

## 2. Overview of Drug Discovery

Early drug discovery towards preclinical studies includes one or more of the following components: hit generation, hit-to-lead expansion, and lead optimization. Hit generation involves screening drug targets against compound libraries (e.g., fragment, scaffold, small molecule libraries, and DELs—DNA-Encoded Libraries) using different methods, the conventional ones being biochemical screening (HTS—High Throughput Screening), biophysical screening (NMR—Nuclear Magnetic Resonance; SPR—Surface Plasmon Resonance; and TSA—Thermal Shift Assay), and computational screening (also known as virtual or in silico screening) [10,11,12,13]. Hit-to-lead expansion and lead optimization involve cycles of medicinal, computational, and synthetic chemistry interwoven with interactive cycles of validating biochemical, biophysical, and cellular assays, as well as structural biology for structure-based drug discovery (SBDD) [14,15], amongst other discovery activities (e.g., ADME/PK, in vivo pharmacology, toxicology, and formulation activities). In many cases, biopharmaceutical companies have also built their own proprietary drug discovery platforms incorporating one or more of these foundational methods with new innovations built on top.

## 3. SaXPy Platform

The SaXPy platform is designed to steer a fragment-based drug discovery (FBDD) and SBDD program and encompasses two technological components: (i) hit generation via high-throughput X-ray crystallography-based screening of a fragment library, one fragment at a time, and (ii) hit-to-lead expansion using computational and medicinal chemistry with analog scoping, scaffold hopping, and fragment growth. Directly using high-throughput X-ray crystallography as a primary screen allows one to immediately assess target engagement via the direct visualization of the hit(s) binding site, binding pose, and protein–ligand interactions. Such information guides the quick advances of hits to functional lead compounds. The SaXPy platform is coupled to tests of in vitro functional activity using target-specific in vitro biochemical assays, guiding the identification of the most effective molecules. Validated compounds obtained using the SaXPy strategy can then be used either as SMIs or as ligands for TPD drugs that target either an orthosteric or non-orthosteric/allosteric site. We describe herein the principles of the SaXPy platform and provide an overview of the first crystal structures of APE1 and POLH bound to small-molecule drug-like fragments, highlighting some of the critical parts of our approach and the results obtained that have enabled us to pursue novel drug development.

## 4. POLH Background

POLH is a member of the Y family of DNA polymerases [16,17,18]. It is a translesion DNA polymerase that can bypass certain blocking lesions, such as those generated via ultraviolet radiation (UVR) or cisplatin, and it is deployed to replicate foci for translesion synthesis (TLS) as part of the DDR. Inherited defects in the gene encoding POLH (*XPV*) are associated with the rare, sun-sensitive, cancer-prone disorder, xeroderma pigmentosum, due to the loss of the ability of POLH to accurately bypass UVR-induced thymine dimers. In standard-of-care cancer therapies that involve platinum-based clinical agents, e.g., cisplatin or oxaliplatin, POLH can also bypass platinum-DNA adducts, negating the benefits of the treatment and enabling drug resistance [19,20,21,22,23,24,25,26,27]. Moreover, POLH has been implicated in resistance to nucleoside analogs, such as gemcitabine and cytarabine [28], and other studies suggest that POLH plays an important role in oxidative stress resistance, likely by carrying out the TLS [29,30] of bulky oxidative base lesions, such as cyclopurines [31,32,33]. Beyond its canonical TLS functions, recent studies found that POLH is important for resistance against the chemotherapeutic alkylating agent, temozolomide (TMZ), seemingly via a TLS-independent mechanism [34]. Finally, POLH has been shown to exhibit the capacity to promote the RNA-templated error-free repair of DNA double-strand breaks (DSBs), a finding that has important implications for carcinogenesis and cancer therapy considerations [35].

POLH overexpression has been linked to the development of chemoresistance in several cancers, including lung, ovarian, and bladder cancers [16]. Consistent with the known biochemistry, elevated POLH expression is correlated with reduced cisplatin sensitivity in models of lung and bladder cancer [19]. The strategic downregulation of POLH in these cases re-sensitizes cancer cells to cisplatin treatment, supporting the targeting of the polymerase in certain situations of acquired drug resistance. The suppression of POLH expression also enhances the cisplatin-induced apoptosis of cancer stem cells isolated from both ovarian cancer cell lines and primary tumors [21]. Furthermore, studies indicate that POLH is a predictive factor for treatment response and the survival of metastatic gastric adenocarcinoma patients receiving oxaliplatin-based first-line chemotherapy [36]. In addition to its well-established role in platin drug resistance, preclinical studies indicate that POLH-deficient cells are 3-fold more sensitive to the nucleoside analogs, B-D-arabinofuranosylcytosine and gemcitabine. POLH-deficient cells are even more sensitive (10-fold) to gemcitabine/cisplatin combination treatment [37], which is a commonly used clinical regimen for treating a wide spectrum of cancers, including bladder, pancreatic, ovarian, cervical, and non-small-cell lung cancers. Additional investigations have revealed that the co-inhibition of POLH and ATR, a protein that is central to the replicative stress response, offers an SL approach for the treatment of a range of cancer types [38,39]. Notably, ATR inhibitors are progressing well in the clinic [40,41,42]. ATR haploinsufficiency, arising due to somatic mutations in one allele, is frequent in certain cancers [43], presenting therapeutic opportunities for POLH inhibition. Very recent work involving functional screening has implicated POLH as an important target for the discovery and development of inhibitors in HORMAD1-positive triple-negative breast cancer (TNBC) [44], a defined disease group that comprises ~60% of TNBC cases, further encouraging the pursuit of POLH inhibitors.

With the value of targeting POLH in the context of new oncology therapeutics, it is not surprising that some attempts have been made in this direction to develop POLH inhibitors: compounds derived from N-aryl-substituted indole barbituric acid (IBA), indole thiobarbituric acid (ITBA), and indole quinuclidine scaffolds [20,45], which are predicted to interfere with template DNA orientation; the computer-aided discovery of a novel class of chromone analogs [46]; and the design of amino acid- and carbohydrate-based compounds [47]. However, these compounds have yet to advance further, and our assessment based on the information available is that this could be due to (i) the precise target engagement/hit validation being unknown due to the absence of crystal structures, preventing further interaction-based optimization, and/or (ii) the suitability of these compounds for further chemistry tractability/optimization.

## 5. APE1 Background

APE1 (also known as APEX1) is a multifunctional protein with a primary function as a DNA repair nuclease and has a separate function as a regulator of transcription factor DNA binding via a redox mechanism [48,49]. As an apurinic/apyrimidinic (AP) endonuclease, APE1 is a central player in the base excision repair (BER) pathway, a process involved in resolving spontaneous, alkylative, and oxidative DNA damage [50]. Specifically, APE1 cleaves at AP sites generated spontaneously either via damage induction or through the action of DNA glycosylases, which initiate classic BER by excising a substrate (often damaged) base moiety [51]. Following the APE1-directed incision event, the remaining 5′-linked abasic fragment is removed, the gap is filled, and the nick is sealed [52]. Due to its prominent role in BER, and since rapidly proliferating cancer cells often upregulate DNA repair enzymes, such as APE1, the protein has emerged as a promising anti-cancer target [53].

Every day, more than 10,000 AP sites are created in each cell under normal metabolic conditions [54], and treatment with some chemotherapeutic agents, as well as ionizing radiation, increases the total number of genomic abasic lesions. If left unrepaired, non-coding AP sites can lead to mutations or replication fork collapse and DSBs that are highly cytotoxic [55]. The importance of APE1 in BER is highlighted by the facts that the enzyme is responsible for greater than 95% of the total AP endonuclease activity in human cells [56] and that the depletion of the protein leads to mammalian cell inviability [57,58], with some selectively for cancer cells [59]. In addition, the siRNA knockdown of APE1 increases the sensitivity of cells to several DNA-damaging agents, most notably alkylators, such as methyl methanesulfonate (MMS) [60,61,62,63]. Thus, APE1 is potentially a relevant therapeutic target for a number of cancers and has been validated in a xenograft model for ovarian cancer, with the knockdown of APE1 greatly reducing tumor growth [64].

APE1 may be particularly relevant in glioblastoma (GBM), for which the TMZ alkylator is the preferred chemotherapeutic agent, as the resistance to TMZ is in part mediated by elevated levels of APE1 [61,65,66,67,68,69,70]. A recent study on exceptional responders to TMZ treatment in GBM, following surgery and radiation, identified the inactivation of APE1 as a source for the exceptionally strong treatment response over 10 years, thus highlighting the promise of APE1 inhibitors in GBM treatment with TMZ [71]. The strategy of combining a DNA-damaging agent with a BER inhibitor, i.e., TMZ, with the PARP inhibitor, veliparib, has been explored in a phase I trial for the treatment of acute myeloid leukemia [72], showing that this combination treatment is well tolerated with efficacy in advanced acute myeloid leukemia. An even more effective strategy might be to employ an APE1 selective inhibitor with TMZ. In addition to the clinical potential of targeting APE1 in combinatorial therapies, it has been demonstrated that APE1 nuclease inhibitors induce the SL of cancer cells that are deficient in DNA DSB repair, i.e., BRCA or ataxia telangiectasia mutated (ATM) defective cell models [73]. The inhibition of APE1 results in the accumulation of DSBs and G2/M cell cycle arrest, presumably due to AP site accumulation and replication fork collapse, ultimately leading to genomic instability and cell death. A similar SL relationship has been observed with APE1 inactivation in PTEN-deficient melanoma cells [74], which likely suffer from analogous DSB repair defects. Another recent study has shown that APE1 inhibition sensitizes cells to inhibitors of DNA-dependent Protein Kinase (DNA-PK) and ATM- and Rad3-related (ATR) kinase, which are both DDR targets of very high therapeutic interest [75]. Thus, clinically effective inhibitors against APE1 nuclease activity have the potential to be used in combination with or as an alternative to DDR inhibitors in the treatment of a range of select DNA repair-deficient cancers. Interestingly, another protein, APE2, which has similarities to APE1 in terms of structure and function, although with a low sequence identity, has also garnered interest as a target for novel DDR therapeutics [76,77,78]. Thus, efforts for the discovery of new and effective APE1 inhibitors can also serve as a foundation for the discovery and development of APE2 inhibitors.

Efforts to date to identify high-affinity, selective small molecule APE1 inhibitors have been met with limited success as evidenced by the few follow-up studies that were conducted to improve the compounds. The published approaches have relied primarily on (i) screening commercially available compounds that were synthesized for other molecular targets, (ii) computational screening, and (iii) pharmacophore modeling [73,74,79,80,81,82,83,84,85,86,87,88,89]. The limited success of these efforts likely stems in part from the inherent difficulty in targeting a protein–nucleic acid interaction [90]. In the case of APE1, the DNA binding site is large, accommodating nine base pairs of duplex DNA, and polar [91,92]. Thus, a new approach is warranted for the discovery of novel and selective APE1 inhibitors.

## 6. SaXPy Platform and POLH/APE1 Hit-Bound Crystal Structures

Prior to our efforts reported herein, there were no available crystal structures of small molecules bound to POLH or APE1 to facilitate SBDD or compound optimization, and there were no experimental structural data on the binding sites or protein–ligand interactions from other published reports of screening and inhibitor development. Towards that end, we have employed our proprietary SaXPy platform, which entails the following steps: (1) isolation of crystallography-grade recombinant protein; (2) optimization of crystallization conditions; (3) fragment library screening, one fragment at a time; (4) determination of fragment-bound protein crystal structures; (5) mapping of the fragment-protein interaction; and (6) hit optimization through medicinal chemistry.

Using previously described protocols [93,94], POLH and APE1 recombinant proteins were purified for X-ray crystallography-based library screening and downstream bioassays. Initial hits were identified by screening a diverse fragment library in a single step using the ABS-OneStep^TM^ crystallography-based library screening platform (Accelero Biostructures, San Carlos, CA, USA) [9] through a partnership. In brief, crystals of apo-POLH-DNA binary complex and apo-APE1 were generated [32,94], employing optimized conditions for reproducibility and scalability to generate several hundred crystals of uniform quality for library screening via ultra-high-throughput X-ray crystallography. Approximately 300 crystals of POLH-DNA or APE1 were used to screen the ABS-Real300^TM^ (Accelero Biostructures, proprietary) 300-fragment library, 1 fragment at a time, typically at a ~10–20 mM fragment concentration and a ~2–4 h soak time. A total of approximately 300 individual X-ray diffraction data sets for each protein target were collected at 100 K using a Pilatus 6M detector (Dectris) at SSRL on beamline 9-2 and the BLU-ICE [95] data collection environment. The data were then processed within the ABS-OneStep platform using XDS [96] and CCP4 [97], with structure determination performed via molecular replacement using our high-resolution apo-POLH-DNA binary complex (1.5Å resolution, which was refined to a crystallographic R/R_free_ of ~13/19%) and apo-APE1 structures (highest resolution of 1.35Å refined to an intermediate crystallographic R/R_free_ of 18/20%) as the search templates. All crystal structures were in the 1.7–2.4 Å resolution range with reasonable crystallographic R/R_free_ values. Due to intellectual property considerations and constraints at the time of publication, the disclosure of the high-resolution details of fragment binding sites and their engagement with POLH and APE1, chemical structures, and other specifics of fragment growth are not shown at this time.

**Figure 1:**
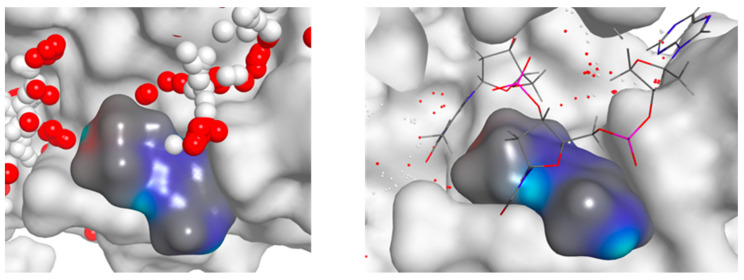
(**left**) POLH Hit 1 site showing the fragment-binding pocket with fragment (blue/gray) and protein (white). The space-filling spheres (red and white spheres) show potential extents of the pocket. (**right**) POLH Hit 1 site with DNA (stick model) visible. Space-filling spheres (red and white dots) show empty pockets.

**Figure 2:**
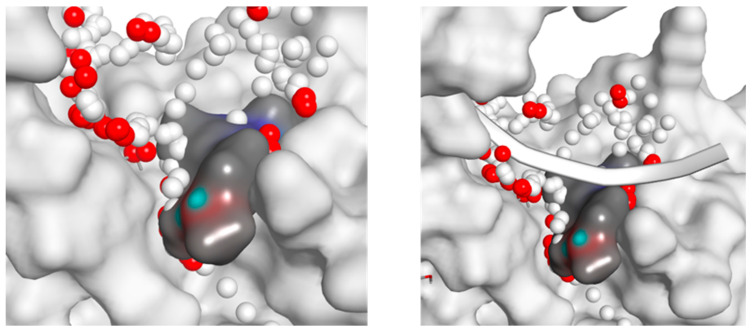
(**right**) POLH Hit 2 site showing the fragment-binding pocket with fragment (blue/gray) and protein (white). Space-filling spheres (red and white spheres) show potential extents of the pocket. (**left**) PLH Hit 2 site with DNA (white ribbon).

POLH Inhibitor Development. For POLH, we obtained a screening hit rate of ~1.3%. Two distinct binding sites were identified on POLH, which we call Hit 1 and Hit 2. Hit 1 is proximal to the orthosteric site, i.e., the DNA binding groove near the polymerase active site. Hit 2 is distal to the orthosteric site and was previously found to be an allosteric site in an in vitro DNA polymerase biochemical assay panel [93,98]. The intrinsic knowledge afforded by our approach was quickly exploited in hit expansion and evolution to advance hits (Figure 1 and Figure 2). An initial small round of hit expansion and medicinal chemistry that included ~40 compounds resulted in inhibitors with a range of functional activity in the in vitro biochemical assay, leading to the rapid identification of inhibitors to advance to subsequent rounds of chemistry to generate a lead compound. A single round of Hit 1 evolution led to one compound with an IC_50_ that was improved by at least 10-fold (XPTx-0289; 230 µM, graph published previously) compared to the ~2 mM IC_50_ of Hit 1, as well as other backup compounds, i.e., about eight compounds with IC_50_ ~1–5 mM and one compound with an IC_50_ of ~8 mM. Hit 2 expansion led to the XPTx-0267 compound with an IC_50_ of ~2 mM. Importantly, our chemical matter is different from the traditional nucleoside analog-based compounds identified in targeting screens for DNA polymerases and are now being pursued in further optimization campaigns.

**Figure 3:**
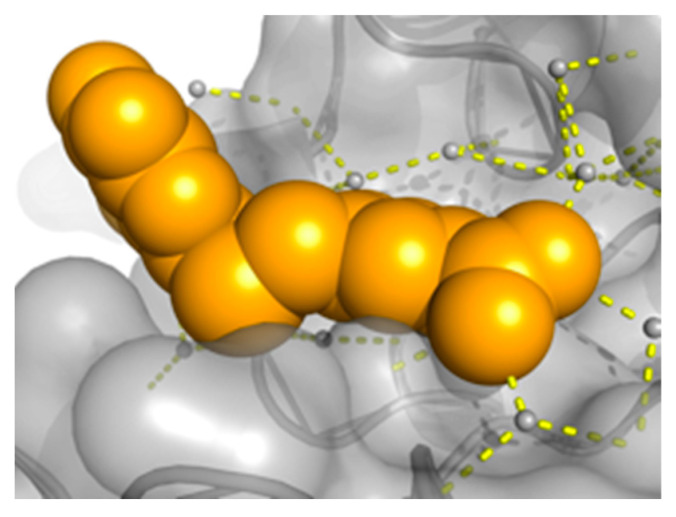
APE1 structure with ligand at the E site (gold spheres).

**Figure 4:**
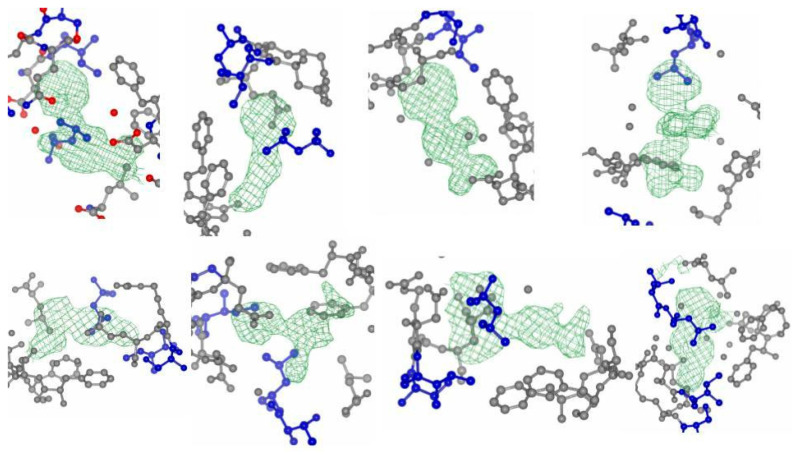
Electron density map of eight APE1 hits at the A site showing the quality of the hits.

**Figure 5:**
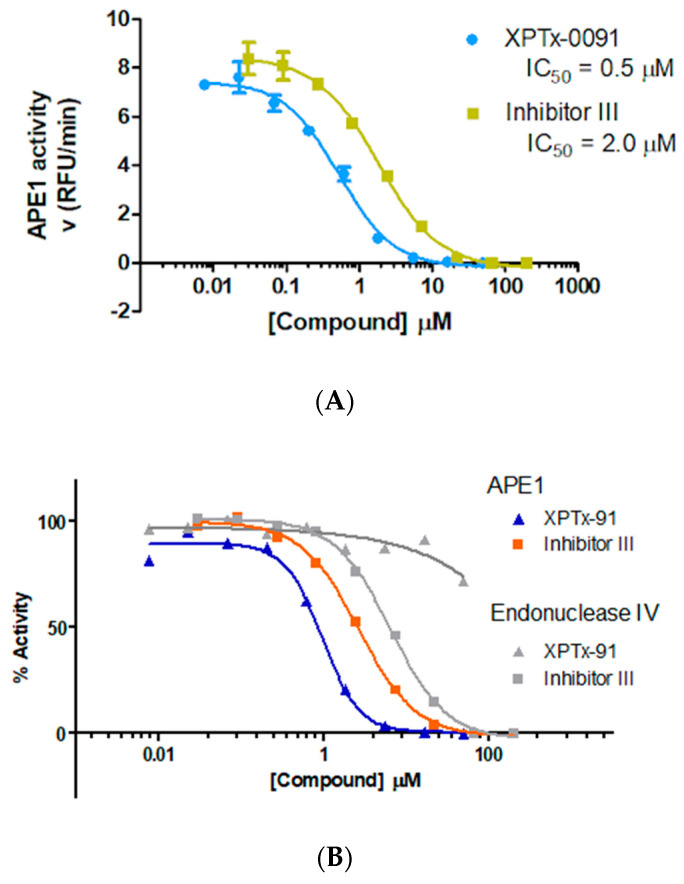
(**A**) IC_50_ of XPTx-0091 and Inhibitor III. APE1 activity was measured as a function of increasing concentrations of XPTx-0091 (blue) or the lead APE1 inhibitor from the literature, Inhibitor III (green). (**B**) Specificity of XPTx-0091 for APE1 over other endonucleases. The IC_50_ for XPTx-001 was determined for APE1 (blue triangles) and EndoIV (gray triangles) using a fluorescence-based endonuclease assay. XPTx-0091 is > 100-fold more selective for APE1 than EndoIV. In contrast, Inhibitor III, the current lead inhibitor from the literature, is only 3-fold more selective for APE1 than EndoIV (orange and gray squares).

APE1 Inhibitor Development. For APE1, we obtained a screening hit rate of ~8%. Hit rates are target-dependent, and the higher hit rate in APE1 compared to POLH reflects differences in the protein size, shape, and solvent-accessible cavities where the ligands can bind. Similar to our POLH case, one of the APE1 binding sites (termed A site) is proximal to the orthosteric site or DNA binding groove (E site), which is where DNA strand incision takes place (Figure 3). We obtained over 20 hits and structures at the A site and show here the quality of 8 hits and corresponding electron density maps of the fragments (Figure 4). Further investigations are pending to understand if this novel binding site might be an allosteric site. Focusing on one of our E site hits, we performed a hit evolution and expansion to ~400 compounds. Using an in-house high-throughput in vitro biochemical assay for APE1 endonuclease activity based on [86], we identified two lead compounds, XPTx-0091 (Figure 5A) and XPTx-0387, with in vitro IC_50_s of ~500 nM and ~250 nM, respectively, as well as several weaker back-up compounds compared to the IC_50_ of the standard APE1 Inhibitor III (~2 µM). XPTx-0091 was also tested for specificity against Endonuclease IV, a non-homologous enzyme with shared AP endonuclease activity, and it was found to have essentially no non-specific activity in this secondary assay (Figure 5B). We then advanced XPTx-0091 to in vitro cell biology assays.

**Figure 6:**
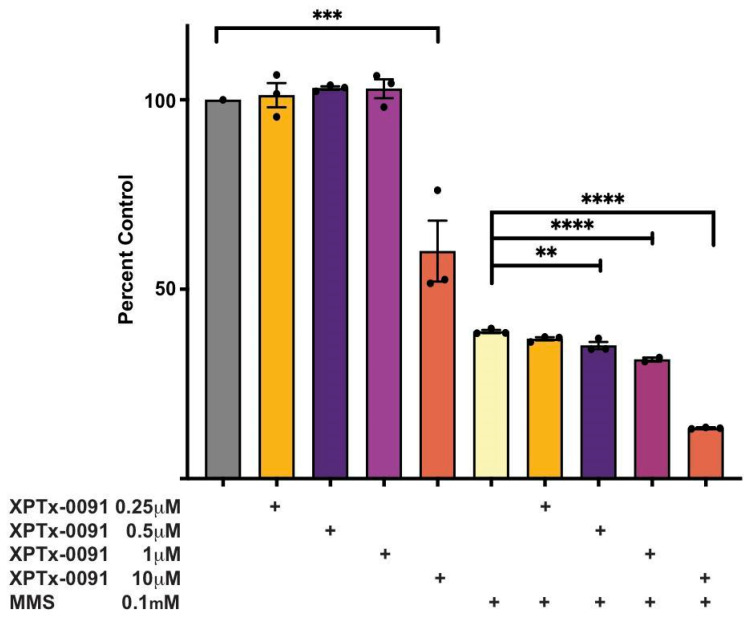
XPTx-0091 increases MMS-induced loss of proliferation. Cells were cultured at 37 °C, 5% CO_2_ in α-Eagle’s MEM supplemented with 10% heat-inactivated fetal bovine serum, glutamine, antibiotic/antimycotic, and gentamicin. The LN428/MPG cell line also required supplementation with G418 (600 mg/mL) (Tang et al., 2011) [101]. Cellular proliferation was determined using the direct cell counting function of the Celigo S imaging cytometer. Statistical analysis was also performed in Prism using one-way ANOVA followed by Tukey’s multiple comparisons test. Data are shown as mean +/− SEM; ** *p* < 0.01, *** *p* < 0.001, **** *p* < 0.0001.

Cells overexpressing the BER-specific N-methylpurine DNA glycosylase (MPG), the enzyme preceding APE1 in BER, exhibit increased sensitivity to DNA alkylators such as MMS, which is likely due to the elevated production of toxic AP sites or DNA strand break repair intermediates. We tested the effects of XPTx-0091 in glioma cells overexpressing MPG (LN428/MPG) in the presence or absence of MMS (Figure 6). The LN428 cell line (originally provided by Ian Pollack, University of Pittsburgh, Pittsburgh, PA) is a glioblastoma-derived cell line that harbors a deletion in both p14ARF and p16, with mutations in the p53 gene [99,100]. Both the parental LN428 cell line and the derivative of LN428 modified for the elevated expression of MPG (LN428/MPG) were described previously [101]. To test the effects of the inhibitor, the LN428/MPG cells were seeded at a density of 1000 cells/well in 96-well tissue culture plates. The following day, the cells were counted using the Celigo S imaging cytometer’s (Nexcelom Bioscience, Lawrence, MA, USA) direct cell counting function. Wells with counts not matching the rest were excluded from the experiments. The wells were then treated with media alone or with media supplemented with 0.1 mM methyl methanesulfonate (MMS) and a dose range of XPTx-0091 (0.25 mM–10 mM) or DMSO as the control. Following 120 h of exposure, the cells were counted as above and graphed using GraphPad (Prism, V9) as the percent control vs. the DMSO-treated cells. XPTx-0091 enhances the cytotoxicity of MMS in a concentration-dependent manner, and this sensitization is observed at concentrations of the compound that are not cytotoxic (i.e., 1 µM; compare purple columns). These observations indicate that the inhibitors of APE1 will likely potentiate the activity of chemotherapeutic alkylators that promote AP site formation without causing broad toxicity.

**Figure 7:**
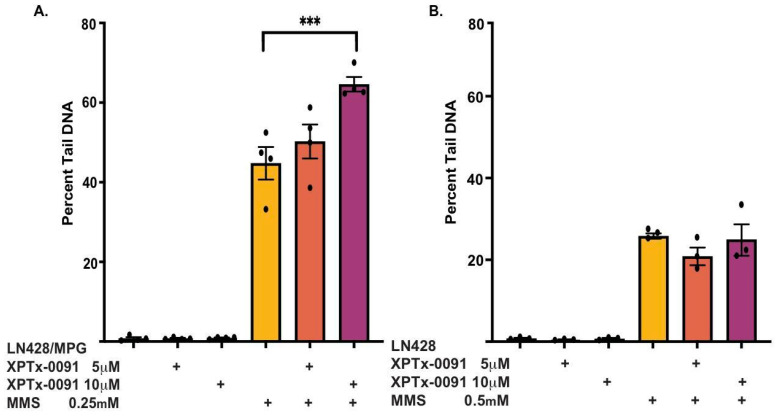
XPTx-0091 increases MMS-induced DNA damage. (**A**) The alkaline CometChip assay was used to quantify the amount of DNA damage in XPTx-0091-treated LN428/MPG cells in the presence or absence of MMS. (**B**) Low-level MPG expressing LN428 cells were also tested using the CometChip assay. In brief, cells were gravity-loaded into 30 µM microwells of the CometChip apparatus for 30 min at 4 °C. After loading, the wells were washed multiple times with PBS and sealed with 0.8% low-melting-point agarose (Topvision, Thermo Fisher Scientific, Waltham, MA, USA, Cat# R0801) in PBS. The CometChip was then submerged in lysis solution with Triton X-100 detergent (BioTechne, Minneapolis, MN, USA, Cat# 4250-050-01) for 2 h at 4 °C and subsequently electrophoresed under alkaline (pH > 13) conditions (200 mM NaOH, 1 mM EDTA, 0.1% Triton X-100) at 22 V for 50 min at 4 °C. After electrophoresis, the CometChip was re-equilibrated to neutral pH using Tris buffer (0.4 M Tris·Cl, pH 7.4), and the DNA was then stained with 1x SYBR Gold (Thermo Fisher Scientific, Cat# S11494), diluted in Tris buffer* (20 mM Tris·Cl, pH 7.4) for 30 min, and de-stained for 1 h in Tris buffer*. Image acquisition was conducted on a Celigo S imaging cytometer (Nexcelom Bioscience, Lawrence, MA, USA) at a resolution of 1 micron/pixel with whole plate imaging to avoid imaging variability. Image analysis was conducted using the dedicated Comet Analysis Software (CAS) v 1.2 (https://casplab-comet-assay-software-project.soft112.com (accessed on 20 October 2023)) with the box size set to 220 × 180 pixels, representing a box size that would capture comets from heavily damaged cells without box overlap. The data acquired were exported to Excel (Microsoft, Redmond, WA, USA) and subsequently to GraphPad (Prism, V9) for statistical analysis. Data are shown as mean +/− SEM; *** *p* < 0.001.

To determine the molecular mechanism underlying the cytotoxicity observed in cells treated with XPTx-0091 (Figure 7), we measured the amount of DNA damage using the CometChip assay (Sykora et al. 2018). This assay measures DNA strand breaks (single and double) as well as alkaline-sensitive sites, e.g., AP lesions, which are converted to strand breaks via β-elimination. The migration of broken or fragmented DNA from the cell nucleus during an electrophoresis step generates a “tail” or “comet”, which can be visualized and quantified using imaging software. For these studies, LN428 and LN428/MPG cells were seeded at a density of 50,000 cells per well in 96-well tissue culture plates. The following day, the cells were treated with XPTx-0091 (5 mM and 10 mM) 30 min prior to +/− 0.5 mM MMS (LN428 cells) or +/− 0.25 mM MMS (LN428/MPG cells) exposure for 1 h. The cells in each well were then trypsinized and transferred to the CometChip apparatus and analyzed for DNA damage as previously detailed [102]. Consistent with our toxicity studies (see above), cells overexpressing MPG that are treated with XPTx-0091 and MMS exhibit a concentration-dependent increase in the DNA damage levels (Figure 7A), while the treatment of cells with XPTx-0091 alone does not result in elevated DNA damage. Additionally, in cells expressing extremely low levels of MPG (Figure 7B), MMS exposure has a lesser effect on the total AP site (DNA damage) levels, which are likely dependent on MPG-mediated base damage excision. Taken together, our data provide strong support for XPTx-0091 targeting APE1 in cells and enhancing alkylator-induced DNA damage and cytotoxicity—the intended mechanism of action of APE1 inhibitors.

## 7. Discussion

Given the success of PARP1 inhibitors in the treatment of certain HRR-defective cancers, fundamental and clinical researchers have been eyeing ways to exploit SL paradigms in the eradication of neoplastic disease. In light of the well-documented changes in the DDRs within cancer cells, coupled with the common use of genotoxins in therapeutic modalities, particular interest, including from a range of pharmaceutical companies, has revolved around the development of novel potent DDR inhibitors. FBDD provides significant benefits in the discovery and development of target-specific novel and active chemical matter, as evidenced by the advancement of several medicines to the clinic [103,104,105,106]. Additionally, SBDD approaches allow for a quick transition from initial hits to lead compounds, with the process being guided by structural insights intrinsic to the method. We describe herein how our SaXPy lead generation platform, which integrates X-ray crystallography-driven fragment library screening, can permit the quick conversion of initial hits to functional lead compounds from even weak hits. As SaXPy allows one to capture novel chemical matter during screening by using the widest detection range method (X-ray crystallography), the approach allows for the discovery of hits irrespective of where they lie on the potency spectrum from weak to strong, whereas a classic biochemical assay cannot typically pick them up, followed by efficient hit-to-lead progression via a structure-guided approach. Our approach simultaneously and uniquely provides experimental information in a high throughput about binding sites; binding poses; protein–ligand interactions; and the separation of hits at orthosteric or potentially allosteric sites and new binding hotspots.

As presented herein, we determined the first X-ray crystal structures of small, novel drug-like compounds bound to POLH and APE1, and rapidly progressed them from initial hit generation to early-stage lead compounds using our SaXPy platform. For APE1, we identified two lead compounds that are now entering lead optimization with in vitro IC_50_s of ~250 nM and ~500 nM. For POLH, while the measured potencies for XPTx-0289 (IC_50_ 230 µM) and XPTx-0267 (2 mM) may appear to be low, such values, and even weaker values, are typical for starting hits in FBDD projects. Recent examples of programs successfully advancing fragments with initial low potencies (>2 mM Kd or IC_50_) include inhibitors against Cyclophilin D [107]; *Mycobacterium tuberculosis* InhA [108]; WDR5-Myc [109]; and our own DDR target, APE1. Notably, in our APE1 effort, the original fragment hit had undetectable activity in the target-specific biochemical assay. The rapid advancement of an initial hit to significantly improved congener inhibitors demonstrates the power of our SaXPy platform to rapidly execute hit-to-lead development campaigns in the design of target-specific inhibitors. Thus, we described herein that SaXPy is applicable to diverse targets, namely POLH and APE1, and that from a single, initial round of fragment growth and expansion, we can rapidly execute hit-to-lead conversion from the experimental knowledge that is intrinsic to the crystal structures.

## Data Availability

Due to intellectual property considerations and constraints at the time of publication, the disclosure of the high-resolution details of fragment binding sites and their engagement with POLH and APE1, chemical structures, and other specifics of fragment growth are not shown at this time.
